# Assessing the satisfaction of health professionals towards the introduction of Maathru Samman pants for normal delivery in the labour room: A multi-site mixed method study from India

**DOI:** 10.1371/journal.pone.0328523

**Published:** 2025-07-28

**Authors:** Venkatashiva B. Reddy, Sirisha Pulla, Anushree Patil, Archana Bhosale, Deepti Tandon, Madhur Verma, Noorin Bhimani, Priti Gupta, Pradeep Aggarwal, Rakesh Kakkar, Star Pala, Wansalan Karu Shullai

**Affiliations:** 1 Department of Community and Family Medicine, All India Institute of Medical Sciences Mangalagiri, Mangalagiri, Andhra Pradesh, India; 2 Head of Division of Clinical Research, ICMR-NIRRCH, Mumbai, India; 3 Department of Obstetrics and Gynecology, Lokmanya Tilak Municipal Medical College and General Hospital, Mumbai, India; 4 Division of Clinical Research, Indian Council of Medical Research (ICMR)-National Institute For Research in Reproductive and Child Health (NIRRCH), Mumbai, India; 5 Department of Community and Family Medicine, All India Institute of Medical Sciences, Bathinda, Punjab, India; 6 Department of Physiology, Lokmanya Tilak Municipal Medical College and General Hospital, Mumbai, India; 7 Centre for Chronic Disease Control, New Delhi, India; 8 Department of Community and Family Medicine, All India Institute of Medical Sciences, Rishikesh, Uttarakhand, India; 9 Department of Community and Family Medicine, All India Institute of Medical Sciences, Bathinda, Punjab, India; 10 Department of Community Medicine, North Eastern Indira Gandhi Regional Institute of Health and Medical Sciences, Shillong, India; 11 Department of Obstetrics and Gynecology, North Eastern Indira Gandhi Regional Institute of Health and Medical Sciences, Shillong, India; Universidade dos Açores Departamento de Biologia: Universidade dos Acores Departamento de Biologia, PORTUGAL

## Abstract

**Introduction:**

Healthcare professionals play a crucial role in the entire childbirth process. In this study, we studied healthcare workers’ response, satisfaction, and demand to use of Maathru Samaan Pants by pregnant women during normal labour.

**Methodology:**

A cross-sectional, mixed-method study was carried in the year of 2023–2024. The study was carried out in four different regions of India. The health care professionals involved in normal delivery where pregnant women used Maathru Samman Pants, were enrolled in the study total of 20 doctors and 39 nurses were studied using a quantitative questionnaire. For the qualitative component, a total of 14 doctors and 16 nurses’ in-depth interviews were conducted. Six FDGs with 29 supporting staff was conducted.

**Results:**

Of the total of 59 healthcare professionals majority of respondents were female, 51 (86.4), and the age range was evenly distributed, with 22(37.3), 21–30 years, 20(33.9), 31–40 years, and 15 (25.4) over 40 years. The majority, 49(83.1), felt that respectful maternity care was extremely important. Most health care staff agreed or strongly agreed that the MSP helped cover their body 58(98.3). A majority found the MSP saved time by preventing dressing/undressing hassle 53(90), and helped with movement before delivery 48 (81.4). A majority, 57 (96.61), agree that the introduction of MSP is a beneficial step towards respectful maternity care. Privacy in both examination and labour rooms is rated highly, with 100% satisfaction across all regions. Most staff support continuing MSP use, 56 (94.91). Some hospitals require additional laundry services or equipment 10 (16.95) or manpower for washing 5 (8.45). Key Themes identified during in-depth interviews and FDGs were comfort, design, acceptability, demand, past experiences, challenges, advantages, funds, reuse, and additional requirements for implementing MSP.

**Conclusion:**

The successful integration of MSPs into healthcare settings can drive innovation and improvements, highlighting India’s leadership in advancing maternal health initiatives.

## Introduction

Healthcare professionals play a crucial role in the entire childbirth process. Their committed and dedicated work in the labour room, providing exceptional care to both mother and child, gives a positive childbirth experience to pregnant women. There is much research that has shown that the presence of highly skilled healthcare professionals in the labour process significantly increases newborn outcomes. [1] There are many recommendations on intrapartum care, which is designed to improve positive childbirth. [2]

Communication-based care provided by healthcare professionals has also been shown to positively influence the childbirth experience and satisfaction among women. [3] Furthermore, promoting interprofessional teamwork among healthcare providers is critical for ensuring positive outcomes during childbirth. [4]

As a healthcare professional dedicated to working in the labour and delivery unit, they have committed themselves to providing exceptional care to women during the childbirth process. Their expertise and compassion play a vital role in ensuring a positive experience for mothers, even in situations where traditional hospital gowns may expose the woman’s body. While some women may feel self-conscious about exposure, their professional demeanor and focus on their care can help them feel more at ease. Offering the option of personalized attire that provides greater coverage is one way to promote their dignity and comfort. This small change can make a significant difference in their overall satisfaction with care.

In many public health facilities, pregnant women often go through labour wearing their own clothes, which are frequently unhygienic since most are unprepared with the necessary items for childbirth. [5] This situation can cause feelings of anxiety and embarrassment. Additionally, many expectant mothers feel uncomfortable in the labour room when men other than their husbands, such as male doctors, nurses, or other health care workers, are present. Recognizing these challenges, this study focused on evaluating the healthcare response, user satisfaction, and demand for Maathru Samaan Pants among pregnant women during normal labour. These pants are designed to improve hygiene, comfort, and dignity throughout the childbirth process, providing respectful maternity care and shy free environment to women in normal labour in public health settings.

## Methodology

### Study settings

A cross-sectional, prospective mixed-method study was carried in the year of 2023–2024. The study was carried out at four different regions of India, which were All India Institute of Medical Sciences, Mangalagiri, All India Institute of Medical Sciences Bathinda, North Eastern Indira Gandhi Regional Institute of Health & Medical Sciences, Shillong, and Lokmanya Tilak Municipal Medical Sciences, Mumbai. Based on the delivery load, medical colleges or district hospitals, community health centers, and primary health centers were considered for data collection. A total of 250 pregnant women were given matthru samaan pants during normal labour at each site, with 200 participants from the district hospital, 30 from the community health center, and 20 from two primary health care centers (10 each). As the health care professionals were involved in every normal delivery conducted in selected hospitals where pregnant women are using these Maathru Samman Pants, their feedback was taken both qualitatively and quantitatively. A minimum of 1 doctor and 2 nurses were interviewed using a quantitative study tool from each health care level from all four sites. The number varied based on the number of health care professionals involved in the labour room for delivery and their willingness to give consent. Finally, a total of 20 doctors and 39 nurses were studied using a quantitative questionnaire. For the qualitative component, in-depth interviews, one doctor and one nurse were considered to study from each health care level at each study site. At the secondary health care level in the north and west sites, doctors’ in-depth interviews could not be conducted due to a lack of their involvement in normal labour. So, a total of 14 doctors and 16 nurses’ in-depth interviews were conducted. For the focus group discussion, supporting staff other than doctors and nurses at the tertiary and secondary health levels involved in the normal labour of women using MSP were considered. However, at the secondary health care level in the west and east, there was only one supporting staff, so FDGs were not conducted. This led to a total of 6 FDGs with 29 supporting staff. The data collection was started on 10 August 2023 and ended 24 May 2024 at south site, in north site it has started on 22 November 2023 and ended on 10 July 2024, In East data collection was started on 16 November 2023 and ended on 22 August 2024 and in west site data collection was started on 9 November 2023 and ended on 10 October 2024.

A written informed consent obtained from doctors, nurses, and supporting staff who are involved in conducting normal delivery using Maathur Samman Pants.

### Study tool

The questionnaire was developed to ensure it covered all relevant topics clearly. First, the key areas related to respectful maternity care and the use of MSP were identified based on the study objectives. Then, questions were drafted for each area, focusing on general information, satisfaction, acceptability, demand, practicality, adaptability, and integration. The questions were designed mostly using a 5-point Likert scale to capture varying opinions, with some yes/no and open-ended questions to gather more detailed responses. After the initial draft, the questionnaire was reviewed by experts to check for clarity, relevance, and completeness. Based on their feedback, the questions were revised and organized into six subsections for easier understanding. Finally, the questionnaire was piloted to confirm that the questions were clear, grammatically appropriate, and easy to understand.

A questionnaire was designed with 45 questions in 6 sub-sections. The first subsection includes general questions, which include name, age, gender, designation, and work experience of the health care professional. This subsection also included questions of respectful maternity care, like the importance of dignity in the labour room, followed by doctors’ and nurses’ knowledge on special dress for delivery, along with the usual practice of birth preparedness requirements during the time of childbirth.

The next subsection is the satisfaction of doctors and nurses for Maathru Samman Pants. The questions were designed using a 5-point Likert scale with options of Strongly Disagree, Disagree, Neutral, Agree, and Strongly Agree. The questions of this section are how MSP helped the pregnant women to cover the body from the waist to the ankle, MSP calf support, and MSP flap help in covering the vulval opening. MSP helps in preventing the hassle of dressing and undressing, MSP helps in moving around the hospital, and the overall usefulness of MSP. After satisfaction, acceptability is the next subsection with a 5-point Likert scale. In these sections, the questions were related to privacy. How MSP prevents the overexposure of the body, MSP use in terms of privacy, shy free environment, and family members’ satisfaction with their pregnant women. Doctors and nurses’ view on recommending Maathru Samman Pants to all pregnant women.

The next section is demand, the questions in this section 4 questions were 5-point Likert scale questions, and 2 yes or no type questions. The questions were designed to address pregnant women’s discomfort in the presence of others, and doctors’ opinions on clothing provided to pregnant women. This section also includes questions related to MSP in comparison to routine delivery dress and how frequently pregnant women ask for MSP, pregnant women preferring the hospital because of MSP, and finally, pregnant women other than study participants asking study participants about dress.

The 4 subsection is practicality; this section is concentrated on the practical feasibility of using Maathru Samman Pants. In this subsection, 7 questions were asked with a 5-point Likert scale, followed by 1 yes or no type question, and one open-ended question. The questions in these sections are MSP help for pregnant women in having multiple birth positions, MSP comfort in conversing to pregnant women, MSP interferences and discomfort in child delivery process and in per vaginal examinations. The sections also included the questions of MSP help in decreasing infections, whether MSP can be washed easily with soap and water, followed by MSP disinfection at their facility, and sterilization of MSP at their facility, along with the number of times the dress can be reused. After practicability, the next subsection is adaptability, which consists of 4 question options with a 5-point Likert scale. The questions in this section are about the amount or space of privacy in the examination room and labour room, whether the process of delivery respects cultural norms, and finally, doctors’ and nurses’ opinions on introducing a personalized delivery attire MSP as a positive initiative.

The last subsection of the questionnaire is integration, which has one question optioned with a 5-point Likert scale, and the remaining 5 questions were open-ended questions. The question is whether the doctors and nurses are interested in continuing the Maathru Samman Pants in their hospital. Remaining open-ended questions are the extra facilities required to implement MSP in their hospital, the sustainability of MSP, and what if their hospital has all the facilities for reusing MSP dress. Finally, the suggestions for changes required in MSP, and are there any pregnant women who refused to use MSP. Of the total questions, 16 questions were negatively phrased and reverse-coded for analysis.

Apart from quantitative components, qualitative data were also collected by conducting in-depth interviews and focus group discussions. From every health care facility, one doctor and one nurse were interviewed. The one focus group discussion was conducted at the tertiary and secondary health care level for all the supporting staff, other than doctors and nurses, who are involved in the labour room and its activities. The qualitative data from the focus group discussions were analyzed following standard procedures for handling transcribed verbatim. First, the audio recordings were transcribed word-for-word to produce accurate textual data. These transcripts were then carefully reviewed and cleaned to correct any errors or unclear sections while maintaining the original meaning. Then the transcripts were translated into English. Next, the data were coded by identifying meaningful segments of text related to the research questions. This coding process was with the help of qualitative data analysis software, grouping similar themes. After coding, the researchers organized these codes into broader categories or themes that represented patterns emerging from the data. Throughout this process, the team regularly discussed and refined the codes and themes to ensure consistency and reliability. Finally, the themes were interpreted in the context of the study objectives, supported by direct quotes from participants to illustrate key points. The Final data is coded and grouped to subthemes like comfort, Design, shy free environment, Acceptability, ease, Desirability, Additional requirements, Past experience, challenges, advantages, reuse, funds which were further arranged in themes like satisfaction, Acceptability, Demand, Adaptation, Practicality and integration in correlation to objectives of the study. Qualitative data analysis was done using ATLAS.ti.8 Educational Single user license.

Microsoft Excel 2019 was used to enter all of the quantitative data, and IBM SPSS Statistics Base v28.0, a Windows-compatible program, was used for analysis. Individual frequencies and descriptive statistics were examined. The mean (SD) was given for continuous variables. The age, gender, and designation of doctors and nurses were the exposure variables. The outcome variables were demand for Maathru Samman Pant, acceptance, satisfaction, practicality, adaptability, and integration among doctors and nurses. Statistical significance was defined as a p-value of less than 0.05.

The relevant data for quantitative analysis are included within the manuscript and its supporting files. All personal identifiers have been de-identified or anonymized. Unique IDs and participant designations have been removed to prevent any potential re-identification and to maintain confidentiality. Since the Primary Health Centers (PHCs) and Community Health Centers (CHCs) involved in the study typically had minimum doctors and nurses, site information has also been excluded as described in the methodology to further minimize any risk of participant identification.

De-identified qualitative transcripts are available upon request due to the sensitive nature of participant designations and the necessity to protect their identities. As data occasionally includes references to sites, cities, or regions during discussions. Requests for access to qualitative data should be directed to the corresponding authors.

## Results

Table 1 represents the distribution of demographic data of doctors and nurses by region of all health care levels. In total, 59 healthcare professionals were surveyed, with 20 (33.9) being doctors and 39 (66.1) being nurses. The majority of respondents were female 51 (86.4), and the age range was evenly distributed, with 22(37.3) between 21–30 years, 20 (33.9) between 31–40 years, and 15 (25.4) over 40 years. Regionally, the South had the highest number of respondents, 18 (30.5), followed by the West, 13 (22), the North, 11 (18.6), and the East, 17 (28.8).

**Table 1:** Distribution of doctors/nurses demographic data by region of all health care levels.

Doctors/nurses demographic data of health care levels										
category	North		South		West		East		Total	
	n	%	n	%	n	%	n	%	n	%
**Age in years**										
**<=20**	0	0.00	0	0.00	2	15.38	0	0.00	2	3.39
**21-30**	2	18.18	3	16.67	10	76.92	7	41.18	22	37.29
**31-40**	6	54.55	6	33.33	1	7.69	7	41.18	20	33.90
**>40**	3	27.27	9	50.00	0	0.00	3	17.65	15	25.42
**Total**	11	100	18	100.00	13	100	17	100	59	100.00
**Gender**										
**Female**	11	100.00	15	83.33	10	76.92	15	88.24	51	86.44
**Male**	0	0.00	3	16.67	3	23.08	2	88.24	8	13.56
**Total**	11	100	18	100	13	100.00	17	11.76	59	100.00
**Designation of doctors/nurses**										
**Doctor**	3	27.27	5	27.78	7	53.85	5	29.41	20	33.90
**Nurse**	8	72.73	13	72.22	6	46.15	12	70.59	39	66.10
**Total**	11	100	18	100.00	13	100.00	17	100.00	59	100.00

Table 2 presents the distribution of doctors’ and nurses’ perceptions of respectful maternity care by region of all health care levels. A total of 59 healthcare professionals were surveyed. The majority, 49 (83.1), felt that respectful maternity care was extremely important, while 8 (13.6) found it very important and 2 (3.4) moderately important. None of the respondents felt it was slightly or not important at all. When asked if they had ever heard of a special dress for delivery, 26 (44.1) said yes and 33 (55.9) said no. Regionally, the South had the highest percentage of respondents who had heard of special delivery dress 18 (77.8), followed by the West 13 (30.8), East 17 (35.3), and North 11 (18.2).

**Table 2:** Distribution of doctors’ and nurses’ perceptions on respectful maternity care by region of all health care levels.

Doctors/nurses perception on respectful maternity care at all health care levels.										
category	North		South		West		East		Total	
	n	%	n	%	n	%	n	%	n	%
**Perceived importance of “Respectful maternity care” while giving birth in their hospital**										
**Extremely Important**	11	100.00	18	100.00	11	84.62	9	52.94	49	83.05
**Very Important**	0	0.00	0	0.00	2	15.38	6	35.29	8	13.56
**Moderately Important**	0	0.00	0	0.00	0	0.00	2	11.76	2	3.39
**Slightly Important**	0	0.00	0	0.00	0	0.00	0	0.00	0	0.00
**Not Important**	0	0.00	0	0.00	0	0.00	0	0.00	0	0.00
**Total**	11	100.00	18	100.00	13	100.00	17	100.00	59	100.00
**Ever heard of a special dress for delivery**										
**Yes**	2	18.18	14	77.78	4	30.77	6	35.29	26	44.07
**No**	9	81.82	4	22.22	9	69.23	11	64.71	33	55.93
**Total**	11	100.00	18	100.00	13	100.00	17	100.00	59	100.00

The data presents satisfaction of doctors and nurses with the use of “Maathru Samman Pants” (MSP) during labour. Most health care staff agreed or strongly agreed that the MSP helped cover their body 58 (98.3), was useful with calf/leg support 52 (88.1), and covered the vulval opening when lying down 59 (100). A majority found the MSP saved time by preventing dressing/undressing hassle 53 (90), helped with movement before delivery 48 (81.4), and was very good in overall usefulness 33 (66.1) and goodoverall usefulness 15(25.42).. The Table 3 presents with overall satisfaction of doctors, nurses across all health care levels (primary, secondary, and tertiary) in different regions regarding the use of MSP for pregnant women during labour. In the North region, the mean satisfaction was 5.00 in all aspects. In the south region the satisfaction scores are high, 4.83 ± 0.41.

**Table 3:** Mean satisfaction of doctors and nurses for Maathru Samman pants in labour room by region at all health care levels. (n = 59).

Satisfaction of doctors and nurses for Maathru Samman Pants										
Category	North		South		West		East		Total	
	Mean	S.d	Mean	S.d	Mean	S.d	Mean	S.d	Mean	S.d
Maathru Samman Pant help a pregnant mother in covering the body from waist to ankle during labour	5.00	0.00	4.83	0.41	4.23	0.44	4.29	0.59	4.58	0.53
Maathru Samman Pant is useful while using calf support and leg support during delivery	5.00	0.00	4.56	0.51	4.23	0.44	3.65	0.93	4.31	0.77
Maathru Samman Pant with a flap on the front help the pregnant mother to cover her Vulval opening, when she was lying on labour table	5.00	0.00	4.56	0.51	4.15	0.38	4.35	0.49	4.49	0.50
Maathru Samman Pant with a flap on the back help pregnant mother to cover her Vulval opening, when she was walking and standing in labour room	5.00	0.00	4.72	0.46	4.23	0.44	4.35	0.61	4.56	0.53
Maathru Samman Pant help you save your time as it prevents the hassle of dressing and undressing while performing per vaginal examinations	5.00	0.00	4.50	0.62	4.15	0.38	3.71	0.85	4.29	0.74
Maathru Samman Pant help the pregnant mother to move around in the hospital before delivery	5.00	0.00	4.28	0.46	3.77	0.73	3.35	1.06	4.03	0.91
Overall usefulness of “Maathru Samman Pant” provided to the PW	5.00	0.00	4.56	1.33	4.77	0.44	4.72	0.46	4.58	0.65

The data shows acceptability of doctors and nurses for Maathru Samman Pants in labour room at all health care levels. 23 respondents (38.98) agreed and 34 (57.63) strongly agreed that the MSP prevents overexposure of private body parts. 6 (10.17) agreed and 21 (35.59) strongly agreed that the MSP increases family member satisfaction. 8 (13.56) agreed and 30 (50.85) strongly agreed that the MSP maintains privacy. 19 (32.20) agreed and 37 (62.71) strongly agreed that the MSP maintains a shy-free environment. 12 (20.20) agreed and 40 (67.80) strongly agreed to recommend the MSP to all pregnant women to maintain privacy. 3 (5.08) agreed and 48 (81.36) strongly agreed to recommend the MSP.The Table 4 presents Total Doctor/Nurses MSP Satisfaction Score had a minimum score of 23 and a maximum score of 35 with mean score of 30.83, with a standard deviation of 3.64.

**Table 4:** Total doctor/nurses MSP satisfaction score all facility by various factors.

Domain	Category	Mean	S.d	t/F statistics	P value
**Site**	North	35	0.01	7.692	<0.001
	South	32.17	2.065		
	West	29.54	2.259		
	East	27.71	3.754		
**Level of hospital**	Tertiary	30.09	4.055	2.072	0.046
	Secondary	31.93	2.556		
	Primary	30.91	3.741		
**Age in years**	<=30	30.25	3.654	0.733	0.69
	31-40	31.13	3.622		
	>40	31.13	3.81		
**Gender**	Female	30.88	3.659	1.293	0.261
	Male	30.5	3.78		
**Perception of importance of RMC in labour room**	Extremely Important	31.56	3.351	0.897	0.543
	Very Important	27.78	3.563		
	Moderately Important	27	1.414		
**Designation**	Doctor	30.7	3.5	3.599	0.001
	Nurse	30.9	3.7		

Table 5 represents the mean acceptability of doctors and nurses for Maathru Samman pants in labour room by region at all health care levels. The data shows from the North, South, West, and East regions, shows high mean scores (4.51–4.69) and relatively low standard deviations (0.47–0.73) across all categories.

**Table 5:** Mean acceptability of doctors and nurses for Maathru Samman pants in labour room by region at all health care levels. (n = 59).

Acceptability of doctors and nurses for using MSP for PW during labour										
Category	North		South		West		East		Total	
	Mean	S.d	Mean	S.d	Mean	S.d	Mean	S.d	Mean	S.d
Maathru Samman pant worn by PW along with a shirt prevent the overexposure of private body parts	5.00	0.00	4.78	0.73	4.00	0.71	4.29	0.47	4.51	0.68
Maathru Samman Pant increased the satisfaction among family members of pregnant women in labour room	5.00	0.00	4.39	1.29	3.92	1.11	3.82	0.53	4.17	1.09
Maathru Samman Pant helped PW in maintaining privacy in labour room during delivery	5.00	0.00	4.67	0.49	4.77	0.44	4.12	0.60	4.59	0.56
Maathru Samman Pant helped in maintaining Shy free environment in labour room during delivery	5.00	0.00	4.78	0.43	4.77	0.44	4.29	0.47	4.68	0.47
Recommend the Maathru Samman Pant along with a shirt to all pregnant women in labour room to maintain privacy	5.00	0.00	4.94	0.24	4.46	1.13	4.41	0.80	4.69	0.73

The Table 6 represents total Doctor/nurse MSP acceptability score had a minimum score of 18 and a maximum score of 35 with mean score 22.64, with a standard deviation of 2.172.

**Table 6:** Total doctor/nurse MSP acceptability Score all facilities by various factors.

Domain	Category	Mean	S.d	t/F statistics	P value
**Site**	**North**	24.64	1.206	8.184	<0.001
	**South**	23.56	1.617		
	**West**	21.92	1.847		
	**East**	20.94	1.919		
**Level of hospital**	**Tertiary**	22.48	2.352	0.753	0.629
	**Secondary**	22.57	1.989		
	**Primary**	22.86	2.167		
**Age in years**	**<=30**	22.45	2.305	0.702	0.67
	**31-40**	22.48	2.129		
	**>40**	23.13	2.125		
**Gender**	**Female**	22.67	2.215	4.331	0.001
	**Male**	22.5	2		
**Perception of importance of RMC in labour room**	**Extremely Important**	23.15	2.021	1.286	0.276
	**Very Important**	20.33	1.414		
	**Moderately Important**	21	0.00		
**Designation**	**Doctor**	22.4	2.2	2.837	0.014

The Table 7 shows 44.07% of respondents always noticed discomfort among pregnant women in the labour room due to the presence of other personnel, while 47.46% often encountered requests for MSP over routine dresses. The MSP is highly rated compared to traditional delivery dresses, with 74.58% rating it very good. Additionally, 57.63% reported that pregnant women prefer their hospital for childbirth due to MSP.

**Table 7:** Distribution of doctors and nurses for the demand of Maathru Samman pants in the labour room at all health care levels. (n = 59).

Demand of Doctors and Nurses using MSP for PW during labour											
Category	n	%	n	%	n	%	N	%	n	%	Total
	Never		Rarely		Sometimes		Very often		Always		
Ever noticed pregnant women feel uncomfortable in labour room in the presence of other personnel	3	5.08	4	6.78	8	13.56	18	30.51	26	44.07	59
You encountered a pregnant woman asking for Maathru Samman pants instead of routine dress during delivery in labour room	8	13.56	6	10.17	10	16.95	7	11.86	28	47.46	59
	**Strongly disagree**		**Disagree**		**Neutral**		**Agree**		**Strongly Agree**		
Agreed mother should be given appropriate clothing in labour room	0	0.00	0	0.00	1	1.69	20	33.90	38	64.41	59
	**Very Bad**		**Bad**		**Moderate**		**Good**		**Very Good**		
Rate the Maathru Samman Pant compared to the routine delivery dress used at your health facility	0	0.00	0	0.00	3	5.08	12	20.34	44	74.58	59
	**Yes**		**No**								
Pregnant women prefer your hospital labour room for childbirth, because of Maathru Samman Pant implementation	34	57.63	25	42.37							59
Other pregnant woman asked them for MSP other than the study participant	17	32.69	35	67.31							52

The Table 8 shows the adaptability of using Maathru Samman Pants (MSP) for pregnant women during labour. A majority 57 (96.61) agree that the introduction of MSP is a beneficial step towards respectful maternity care. Privacy in both examination and labour rooms is rated highly, with 100% satisfaction across all regions. Additionally, respecting cultural and organizational goals during delivery is deemed very important or extremely important by 58 (98.31) of respondents.

**Table 8:** Distribution of doctors and nurses for adaptability of Maathru Samman pants in the labour room by region at all health care levels. (n = 59).

Adaptability of using MSP for PW during labour										
category	North		South		West		East		Total	
	n	%	n	%	n	%	n	%	n	%
	**Agree/Strongly Agree**									
Introduction of MSP is a positive initiative for Respectful maternity care.	10	90.91	18	100.00	13	100.00	16	94.12	57	96.61
	**Good/Very Good**									
How would you rate the amount of space and facilities to maintain privacy in the examination room?	11	100.00	18	100.00	13	100.00	17	100.00	59	100.00
How would you rate the amount of space and facilities to maintain privacy in the labour room?	11	100.00	18	100.00	13	100.00	17	100.00	59	100.00
	**Very Important/Extremely Important**									
Undergoing the process of delivery in a way that respects our culture and organizational goal, Would you say it is:	11	100.00	18	100.00	13	100.00	16	94.12	58	98.31

The Table 9 shows majority 49 (83.05) agree that MSP facilitates various birth positions, with North and South completely in agreement. MSPs were supported for being easily washable 45 (76.27), disinfectable 52 (88.14), and sterilizable 56 (94.92).

**Table 9:** Distribution of doctors and nurses for practicability of Maathru Samman pants in the labour room by region at all health care levels. (n = 59).

Practicability of using MSP for PW during labour										
Category	North		South		West		East		Total	
	n	%	n	%	n	%	n	%	n	%
	Agree/Strongly Agree									
**Maathru Samman pant (MSP) helps the mother to give birth in different positions**	11	100.00	18	100.00	12	92.31	8	47.06	49	83.05
**sterile Maathru Samman Pant (MSP) and a shirt help in decreasing infections to mother and baby along with all clean practices in labour room**	11	100.00	17	94.44	10	76.92	14	82.35	52	88.14
**felt comfortable while doing Per Vaginal examinations, and procedures and conducting the delivery of baby in pregnant women wearing Maathru Samman Pant (MSP) in labour room**	6	54.55	18	100.00	9	69.23	7	41.18	40	67.80
	Always/Very often									
**MSP is easily washable with water and soap at your facility**	8	72.73	16	88.89	8	61.54	13	76.47	45	76.27
**MSP can be disinfected easily at your facility**	10	90.91	18	100.00	10	76.92	14	82.35	52	88.14
**MSP can be sterilized using an autoclave at your facility and reused**	11	100.00	18	100.00	12	92.31	15	88.24	56	94.92
	>40 times/Always									
**How many times MSP dress can be reused**	0	0.00	6	33.33	5	38.46	7	41.18	18	30.51
	Yes									
**Maathru Samman Pant along with a shirt helped to feel comfortable conversing with the pregnant mother during the procedure and delivery of the baby in labour room**	11	100.00	18	100.00	12	92.31	17	100.00	58	98.31
**MSP interfere during the child delivery procedure or Per Vaginal examination**	0	0.00	0	0.00	0	0	0	0.00	0	0.00

Table 10 shows across all healthcare levels, most staff support continuing Maathru Samman Pants (MSP) use 56 (94.91). Some hospitals require additional laundry services or equipment 10 (16.95) or manpower for washing 5 (8.45). Minor suggested improvements include larger vulval sizes 4 (6.78), 3 injection holes (5.08), and material modifications 4 (6.78).

**Table 10:** Distribution of doctors and nurses for the Integration and expansion of using Maathru Samman pant in the labour room by region at all health care levels.

Integration and scope for expansion of using MSP for PW during labour									
Category	North		South		West		East		Total
	n	%	N	%	n	%	n	%	N
No. of Doctors/Nurses agreed that they want to continue the Maathru Samman Pant use in their hospital	11	19.64	18	32.14	11	19.64	16	28.57	56
No. of Doctors/Nurses expressed that hospital need any extra facility to implement Maathru Samman Pant (n = 15)									
Laundry services/equipment’s	0	0.00	3	30.00	2	20.00	5	50.00	10
Manpower for washing MSP	0	0.00	4	80.00	0	0.00	1	20.00	5
No. of Doctors/Nurses expressed that Maathru Samman Pant’s Implementation is sustainable due to following reasons *									
Acceptability (n = 52)	10	19.23	16	30.77	9	17.31	17	32.69	52
Privacy (n = 8)	2	25.00	3	37.50	0	0.00	3	37.50	8
Comfort (n = 8)	6	75.00	1	0.00	0	0.00	1	12.50	8
Shy free environment (n = 5)	5	100.00	0	0.00	0	0.00	0	0.00	5
Desirability (n = 9)	0	0.00	1	11.11	6	66.67	2	22.22	9
No. of Doctors and Nurses agreed that hospital have all facilities for reusing Maathru Samman Pant (n = 44)	11	25.00	11	25.00	11	25.00	11	25.00	44
Changes needed in Maathru Samman Pants to improve usefulness									
None (n = 45)	9	20.00	17	37.78	10	22.22	9	20.00	45
Increase Vulval Size of MSP (n = 4)	2	50.00	0	0.00	0	0.00	2	50.00	4
Hole at thigh region of MSP for injection (n = 3)	0	0.00	0	0.00	0	0.00	3	100.00	3
Others (Colour, increase flap size, Need ties or elastic and quality of cloth) (n = 4)	0	0.00	1	25.00	0	0.00	3	75.00	4

*multiple option

### Qualitative

Table 11 details Themes, subthemes, and codes of qualitative interviews. For in-depth interviews, there were 30 participants, 14 (47) doctors and 16 (53) nurses, interviewed in total, with 6 (20) males and 24 (80) females. In the North, all 7 were female and no males. The South and East had 2 (25) males and 6 (75) females. The West had 2 (29) males and 5 (71) females. In the 21–30 years age group, there were 13 (43) participants. For ages 31–40 years and 40 years of age, there were 11 and 6 participants, respectively.For the Focus Group discussion, 29 supporting staff were studied, who were all female.

**Table 11:** Showing themes, subthemes, and codes of in-depth interviews.

Theme	Sub theme	Code
Satisfaction	Comfort	Comfortable for PW to move around
		MSP was comfortable to BC
		MSP was comfortable to PW
		MSP was Comfortable to staff
	Design	Dress ease
		Need buttons for flaps
		Staff view on dress design
		Zips/ buttons for flaps and holes
	Shy free environment	Dress provides privacy
		Prevents exposure of entire body
		Respectful environment
		Shy free environment for delivery
Acceptability	Acceptability	BC Satisfaction for MSP
	Ease	Easy to convenience PW
		Health care benefit to PW
		Infection free delivery
		Infection rate decrease
Demand	Desirability	MSP is helpful
		MSP success
		No challenges with dress
		No hindering factors
		No rejection for dress
		Easy to implement
		Expression of interest of staff
		less time required to convenience PW
		PW preference for MSP
		PW satisfaction for MSP
		Staff intent to continue the dress
		Staff view on community acceptance
Adaptation	Additional Requirements	Dresses requirement
		Equipment required for cleaning
		Facilities required for maintenance
		Funds/ man power required for cleaning
		Funds required to maintain
		Staff explanation on dress to PW
		Staff need counselling
		Staff support for cleaning
		Studies to prove
	Past experience	Available/ Regular dress for delivery
		Comparison to available/ regular dress
Practicality	Challenges	Challenge with Reuse
		Challenges in complicated cases
		Change of dress material
		Disposable dress
		Hindering factors
		Uncomfortable for PW to use washroom
	Advantageous	Dress Increases warmth to baby
		Dress is convenient for taking photos
		Dress provides warmth
		MSP promotes hygienic environment
		Prevents Infections
		PW is Cooperative due to MSP
		Unique dress code
	Reuse	Comfortable to reuse
		Process for Reuse
		Reuse of dress
Integration	Funds	CDS provides everything
		Funding sources
		Government Providing everything
		No Extra funding needed
		No funding source
		No New facilities required
		Staff view on further implementation
		Staff view on government implementation
		Support to MSP by central/state

### Theme: Comfort

Staff found it comfortable to use specialized dresses for pregnant women during normal delivery, as these dresses effectively prevented overexposure of the body. This not only ensured a higher level of modesty and dignity for the patients but also made it easier for the staff to maintain professional and comfortable working conditions during the delivery process.

#### Tertiary health care level.

*“CandidateS-DH-D-01: Those who wore this dress are very cooperative during delivery because they are shy free. Pregnant women liked delivery in this dress.” “Candidate 3 S-DH-FGD: The dress is loose and nice. For them to move here and there and to use the bathroom there is no discomfort.” “Candidate 3 N-DH-FGD: This dress is very nice. There is no discomfort with wearing this dress. Due to this dress, patients feel comfortable in moving around the ward and also during breastfeed. This dress has lots of benefits. So, every pregnant woman likes to use this dress.” “Candidate NE-NEI-DC-200: Okay so the thing is,it has one advantage. The clothes that we give are sterile, clean and neat and tidy. Plus, there is adequate coverage especially during winter. The mother feels confident and comfortable that she’s completely covered. So in that way, it is quite different and advantageous.” “Candidate 3 W-LT-PW-FGD: With Maathru Samman pants patients can move freely in the ward they can go to the washroom also.”*

#### Secondary health care level.

*“Candidate 4 S-CHC-FGD: The pregnant women are very satisfied with the dress. By wearing this dress their entire body will not get exposed. It is very helpful for them to move here and there.” “Candidate 4 N-CHC-FGD: Mam, I do not think this dress needs any changes. This dress covers the entire body of patients. So, patients feel comfortable. Also, birth companions feel good to see patient’s happy and comfortable.” “Candidate NE-CHCM-DC-230: I think patients will accept MSP. They will feel like they are treated in a hospital and will also feel taken care of.” “Candidate W-CHC-SN-01: Yes, it is definitely different from regular clothes on regular clothes on regular uniform and it is comfortable during deliveries of baby, and it is really comfortable during PV examinations.”*

#### Primary health care level.

*“Candidate S-PHCT-D-01: Yes, because after using the delivery dress we could take Aarogyasri photos and for others the dress is convenient. The birth companion is also comfortable. It is like a uniform and it is nice.” “Candidate N-PHCK-SN-01: Birth companions feel good and they have no issue with this dress because the patients feel more comfortable in this dress.” “Candidate NE-PHCS-DC-240: One advantage is that it maintains uniformity. Another one is the privacy that it gives for the patients. It also prevents the hassle of getting dressed and undressed too many times during labour. Its convenience increases especially during winter. We don’t have to lift up their clothes anymore because an opening is already available in the pants and we can cover it with the flap attached there. Since our place is extremely cold, patients feel more warm while wearing MSPs.”*

### Theme: Design

Staff have expressed positive views on the design of the dress, particularly appreciating the flaps that cover the openings through which the baby is delivered, ensuring privacy and dignity. They have also suggested additional design requirements for the dress to better suit pregnant women during normal delivery. They have highlighted the necessity for zippers or buttons at feeding holes and for the flaps.

#### Tertiary health care level.

*“Candidate 5 S-DH-FGD: The dress you have provided prevents the exposure of the body. It was made to expose only those body parts that are required for delivery. During their delivery even if any gents entered the labour room they are not shy and the dress also has excessive cloth which is used for covering.”*

*“Candidate N-DH-SN-01: This dress is nice but if the vaginal cut of this dress is in U shape, then this dress is more comfortable for patients. This is overall what I want to suggest.”*

*“Candidate 3 N-DH-FGD: Yes, if patients wear Maathru Samman Pant dress then doctors and nurses also feel good and help in easy delivery.”*

*“Candidate W-LT-SN-01: This dress is helpful during conducting. This dress maintains privacy and this dress has two flaps which helps to reduce unwanted exposure during conducting.”*

#### Secondary health care level.

“*Candidate S-CHC-SN-01: This MSP dress has thread to tie and it is easy to use. It is good for feeding immediately after delivery.*”

*“Candidate 3 N-CHC-FGD: No, there is no discomfort for pregnant women to wear this dress. This dress is very comfortable for moving here and there as extra fabric is added to this dress.”*

*“Candidate NE-CHCM-DC-230: We don’t have any challenges however, it would help if they can alter the pants a little. They can add additional ties at the thigh region to help fix the pants so that it does not interfere during delivery.” “Candidate*

#### Primary health care level.

*“Candidate S-PHCT-D-01: Yes, I strongly recommend it would be good if some buttons were provided for flaps. The zip should be provided for the top for breastfeeding as it does not get soiled. Nowadays it is available for nighties.”*

*“Candidate N-PHCB-D-01: This dress is comfortable and easy to use for patients.”*

*“Candidate NE-PHCS-DC-240: Firstly, these MSPs are easy to put on and protect the privacy of the mother. At the same time, it prevents the patients from getting dressed and undressed too many times because we can use the flaps instead of taking off everything.”*

### Theme: Shy free environment

The introduction of personalized dresses for normal delivery has fostered a shy-free environment for staff, as it effectively prevents the exposure of pregnant women during the delivery process. This ensures that staff can work comfortably and confidently, knowing that the dignity and privacy of the pregnant women are maintained. By providing a shy-free environment, the personalized dress enhances the overall experience for both staff and pregnant women, promoting professionalism and respect throughout the delivery process.

#### Tertiary health care level.

*“CandidateS-DH-D-01: In the past the dress they used needed to be removed entirely. But in this dress, Unnecessary parts are not exposed by using this dress and only the required body parts are exposed. so, this MSP dress is very useful.”*

*“Candidate S-DH-SN-01: This dress prevents the overexposure of the entire body and also it exposes only those body parts that are involved in delivery procedure.”*

*“Candidate N-DH-D-01: Definitely, due to this dress the population of patients will increase. Because patients need privacy and as this dress maintains privacy and gives comfort. So, I think the population will increase.”*

*“Candidate NE-DH-SN-200: We find it helpful because in place of gynae sheet we can substitute it with MSPs. It has given more privacy for patients as well.”*

*“Candidate W-LT-SN-01: Do maintain privacy. To give a shy free environment during conducting. To give better satisfaction to the patient.”*

*“Candidate W-LT-SN-01: This dress is helpful during conducting. This dress maintains privacy and this dress has two flaps which helps to reduce unwanted exposure during conducting.”*

#### Secondary health care level.

*“Candidate S-CHC-D-01: This dress covers the entire body. By wearing this dress it provides a shy free environment for pregnant women such that everyone likes to use this MSP dress.”*

*“Candidate 3 N-CHC-FGD: This dress is very nice. This dress covers the entire body and prevents the unnecessary exposure of the body. So, it maintains complete privacy easily.”*

*“Candidate NE-CHCM-DC-230: One positive aspect of MSP is that it is hygienic and cleaner compared to the patient’s clothes. Another advantage is that we can easily access the vaginal area and conduct delivery. It also helps to protect the patients’ privacy.”*

#### Primary health care level.

*“Candidate S-PHCT-D-01: We will already change the dress to pregnant women after entering the labour room. This dress is comfortable for every delivery. It prevents the exposure of the abdomen.”*

*“Candidate N-PHCK-SN-01: Overall this dress is very good. Patients as well as their birth companions feel good. They prefer to use this dress over normal clothes. This dress is comfortable and maintains privacy.”*

*“Candidate NE-PHCS-DC-240: Firstly, these MSPs are easy to put on and protect the privacy of the mother. At the same time, it prevents the patients from getting dressed and undressed too many times because we can use the flaps instead of taking off everything.”*

*“Candidate W-PHC-G-DR-01:Yes it is good and new idea, so we can provide care for mothers, respect and her privacy will be maintained.”*

### Theme acceptability

The specialized dress for normal delivery has achieved high acceptability among staff, being preferred by pregnant women and satisfactory to both them and their birth companions. Due to the positive feedback and enhanced comfort for all parties involved, staff intend to continue using the dress. They also support further implementation by the government, recognizing the potential for widespread benefits in maintaining dignity and comfort during the delivery process.

#### Tertiary health care level.

*“CandidateS-DH-D-01: Yes, the pregnant women and their attender are very happy. The labour room had less infection. Prior Episiotomy and wound infections are more. When the pregnant women were changed to MSP dress the infection rate decreased.*

*“Candidate N-DH-D-01: Yes, this dress can develop and improves the health care delivery system in India. On basis of patients perspective and patients satisfactory outcomes, this dress is very beneficial. So, definitely this dress will improve the delivery system in India. ”*

*“Candidate NE-DH-SN-200: We find it helpful because in place of gynae sheet we can substitute it with MSPs. It has given more privacy for patients as well.”*

*“Candidate W-LT-D-01: This dress I have seen for the first time in our hospital. ICMR project arranges this dress. This dress is Used for normal delivery patients. This dress is different and it is helpful for per vaginal examination.” “*

#### Secondary health care level.

*“Candidate S-CHC-SN-01: Everyone is comfortable with this dress. I think everyone will support this dress.”*

*“Candidate 4 N-CHC-FGD: We showed them the dress and then we explained the benefits and features of this dress. After listening to this they become ready to wear this dress. ” “Candidate NE-CHCM-DC-230: Yes it will be beneficial as the risk of infections will decrease if we sterilize these MSPs with proper technique.”*

*“Candidate NE-CHCM-DC-230: MSP has reduced the risk of infections because we don’t know where the patients come from or what they were doing before coming here. So it is better to have them change into MSP in order to maintain sterility during the whole procedure.”*

#### Primary health care level.

*“Candidate S-PHCT-SN-01: Using this dress for delivery the birth companion who is beside the pregnant woman was satisfied.”*

*“Candidate N-PHCK-D-01: Yes, as I already said these dresses are more comfortable, easy to handle, easy maintenance and also contamination rate is low. ”*

*“Candidate NE-PHCJ-DC-250: First of all, it is very easy for patients to get dressed and it is also easy for us to conduct delivery. It is also easier for us to maintain aseptic conditions during the time of childbirth.”*

### Theme: Demand

Maathru Samman pants are a specialized dress designed for women undergoing normal delivery. MSP aim to preserve the dignity of the woman by minimizing body exposure during childbirth, even in the presence of a birth companion. Additionally, they help in maintaining a sterile environment to prevent infections. The demand from doctors for such innovative maternity wear underscores its perceived benefits in promoting comfort, privacy, and hygiene during the birthing process.

#### Tertiary health care level.

*“CandidateS-DH-D-01: Government has made a good initiative. For the safety of mother and newborn the government is implementing new ideas among them. I feel this MSP is also a good one.”*

*“Candidate N-DH-SN-01: According to me, the population of patients will definitely increase because after going home, ladies in their villages talk about this dress. So this dress reduces the anxiety of pregnant women.”*

*“Candidate NE-NEI-DC-200: Here most of the patients agree, I think they would prefer MSPs.”*

*“Candidate NE-DH-SN-200: As long as the supply is available, we will continue using them.”*

*“Candidate W-LT-D-01: Feeling better while using Maathru Samman Pants.”*

#### Secondary health care level.

*“Candidate S-CHC-D-01: In the labour room it will be helpful to use for pregnant women without any discomfort. For us it is good to visualize. So, I think it should be introduced in every labour room.”*

*“Candidate N-CHC-SN-01: No one will complain ever regarding any challenges till now. In fact everyone was praising this dress. ”*

*“Candidate NE-CHCM-DC-230: We have an intent to continue”*

#### Primary health care level.

*“Candidate S-PHCP-SN-01: As the dress covers the patient’s body completely. So, they prefer to use this dress only.”*

*“Candidate N-PHCK-D-01: I would say I will try my best to encourage each and every patients and their relatives to wear this pant. I would really like to encourage other government and also private health facilities to use this dress. ”*

*“Candidate NE-PHCJ-DC-250: Of course they will continue using these MSPs.” “Candidate W-PHC-G-DR-01: Yes it is different as we can do PV easily while wearing dress and delivery is also easy in wearing dress.”*

### Theme: Past experience of doctors and nurses

Traditional routine dress for normal delivery worn by pregnant women often involved minimal coverage, with hospital gowns that left much of the body exposed. Experienced staff are familiar with these gowns, which, while functional for medical examination and interventions, could make both patients and staff uncomfortable. The exposure sometimes caused discomfort among staff, who are trained to maintain professionalism but are also empathetic to the patient’s feelings of vulnerability.

#### Tertiary health care level.

*“Candidate S-DH-SN-01: Till now we have a gown and dress which has threads on the backside.”*

*“ Candidate N-DH-SN-01: According to me, these dresses are different from normal routine clothes because firstly these dresses are easy to wear and secondly these dresses maintain privacy. Also, patients feel more comfortable during breastfeeding. In normal clothes, patients face many difficulties. So these dresses are very effective in this way.*

*“Candidate W-LT-SN-01: As compared to routine dress this dress is very comfortable for the patient. In routine dress there is unwanted exposure of other body parts during conducting.”*

#### Secondary health care level.

*“Candidate S-CHC-D-01: In prior they have provided gown for the delivery. The gown has an opening in the back which is not comfortable to the patient.”*

#### Primary health care level.

*“Candidate S-PHCT-D-01: Yes I suggest, because pregnant women are coming in different ways. Some are wearing churidars, sarees and in nighties. Actually all those attires are uncomfortable for conducting delivery. This dress is simple when visitors and birth companions visit. I suggest this dress is very good.”*

*“Candidate NE-PHCS-SN-240: The difference between the normal delivery clothes and MSP is that it has a specific opening to allow access to the perineal area. It also has a precise space for breastfeeding. MSPs are quite convenient*

### Theme: Additional requirements

The introduction of a specialized cloth dress for normal delivery requires addressing some additional requirements. These include dedicating time to explain the dress to patients, securing funds for maintaining the dresses and ensuring a sufficient stock to meet ongoing needs. These steps are essential to ensure the successful implementation and upkeep of the specialized dress system.

#### Tertiary health care level.

*“CandidateS-DH-D-01: Actually the quantity needs to be increased to help with the delivery load. If it’s the rainy season some pants may not be dried so I think the quantity can be increased.”*

*“Candidate 1 N-DH-FGD: But it is good if a separate washing machine is provided.”*

*“Candidate NE-NEI-DC-200: In government facilities, the number of patients is very high. So at times we will have to have an adequate set of clothes and get it sterilized every time, so that can be an issue. So because of the rush we might not have a set of clothes (MSP) to put on every time for each and every patient because the staff is quite busy. So other than that it would be fine.”*

#### Secondary health care level.

*“Candidate S-CHC-SN-01: It would be good if more MSP dresses were provided. There should be sufficient availability even in the rainy season.”*

*“Candidate N-CHC-SN-01: We want more MSP dresses and working staff if this dress is implemented on area. Till now, we do not require any fund but if we will aware the people by posters, banners and camp then we need some additional fund for this. ”*

*Candidate NE-CHCM-DC-230: They can help by increasing the supply of MSPs and also help with the wages for the support staff.”*

*“Candidate W-CHC-SN-01: Factors like economic factors & manpower for washing.”*

#### Primary health care level.

*“Candidate S-PHCT-D-01: It would be good if provided with dhobi because we have many other Linen’s also along with this dress. So we can conduct smooth deliveries if dhobi was provided. We should also get washing materials.”*

*“Candidate N-PHCB-D-01: We require these dresses in high quantities. ”*

*“Candidate NE-PHCS-SN-240: The factor which makes the implementation of MSPs very difficult in our PHC is the lack of staff in the laundry department. We also face a problem regarding sterilization of clothes. We use a small autoclave machine in which we sterilize cotton and gauze pieces.*

*“Candidate W-PHC-G-SN-01: Clean washing area and expenses should be given to necessary items required to it.”*

### Theme: Reuse

The reuse of cloth-made dresses for pregnant women during normal delivery provides enhanced modesty and comfort by preventing unnecessary exposure of body parts. These dresses are designed to ensure privacy while allowing easy access for medical procedures. Made from breathable, natural fabrics, they offer a sustainable and cost-effective alternative to disposable gowns, ensuring a dignified and eco-friendly childbirth experience.

#### Tertiary health care level.

*“Candidate S-DH-SN-01: The dress will be washed in a bleach solution and after drying it will be autoclaved (for reuse).”*

*“Candidate NE-DH-SN-200: We can manage these MSPs by reusing and recycling.”*

*“Candidate W-LT-SN-01: This dress can be used many times.it found helpful during conducting (delivery).”*

*“Candidate 2 W-LT-FGD: After washing and autoclave this dress can be reused.”*

#### Secondary health care level.

*“Candidate 6 S-CHC-FGD: The dress you have provided is very good for reusing again and again.”*

*“Candidate NE-CHCM-SN-230: After every delivery, we change the clothes of patients into clean ones. So these gowns that are soiled are sent to the support staff to wash and autoclave them. In this way we get to reuse it.”*

*“Candidate W-CHC-SN-01: Here autoclave is not possible but we can wash the clothes everyday & proper use of clothes. ”*

#### Primary health care level.

*“Candidate S-PHCT-SN-01: The dress will be cleaned here using antiseptic solution and after drying it will be autoclaved (for reuse).”*

*“Candidate W-PHC-G-DR-01:We wash the clothes and re-use them.”*

### Theme: Challenges of doctors and nurses

Every new introduction inevitably comes with its own set of challenges or limitations. Similarly, the introduction of Maathru Samman Pants made staff encounter some hurdles. These included difficulties in cleaning the dress, the time required to explain its purpose to pregnant women, securing adequate funding, patients’ refusal to reuse the dress, and the discomfort pregnant women felt while using washrooms. Addressing these issues is crucial for the successful adoption and acceptance of the new dress by both staff and patients.

#### Tertiary health care level.

*“Candidate S-DH-SN-01: Dresses are used again and again by washing them along with an autoclave. But the pregnant women may not prefer to reuse this dress as it was already used by other pregnant women.”*

*“Candidate NE-DH-SN-200: While we still have enough time to change patients into MSPs, we are aware of its convenience but when they are fully dilated, we do not change patients into MSPs anymore. Another disadvantage is if patients wear these MSPs and do not lie still, this will make it difficult to see the vulval opening. Furthermore, when giving IM injection, a hole near the thigh area should be there. This is because when giving IM injections we have to take off their pants or roll it up to the thighs.”*

#### Secondary health care level.

*“Candidate S-CHC-D-01: This dress needed to be washed and autoclaved for reuse. The patients may not be likely to use one dress which was used by other patients.”*

*“Candidate NE-CHCM-DC-230: The problem is that we are not regular in terms of using these MSPs. At times, patients arrive when they are fully dilated so we don’t have time to change their clothes. If patients arrive before active labour then we have time to prepare and change their clothes.”*

*“Candidate W-CHC-SN-01: I think the challenges are its not comfortable for walking while using washrooms.*

#### Primary health care level.

*“Candidate S-PHCT-SN-01:: I feel it would be good if the dress is disposable.”*

*“Candidate N-PHCK-SN-01: Yes ma’am, we are using this dress for patients but we don’t have any proper washing facilities. So, we convinced family members of patients to wash the dress properly and return the dress to the hospital as soon as possible.”*

*“Candidate NE-PHCJ-SN-250: One problem will be if patients refuse to wear it. Another problem is the complaints regarding the material of the MSPs. The fabric that is used is very difficult to wash and remove blood stains. If the fabric is similar to scrubs then it will be easier to clean.*

*“Candidate W-CHC-SN-01: I think the challenges are that it is not comfortable for walking while using washrooms.”*

### Theme: Advantages

Apart from ensuring respect and privacy, the specialized dress for normal delivery is also comfortable for pregnant women. Staff observations highlight that the dress provides warmth to both mother and baby and helps prevent infections. These features make it a unique code of dress for those undergoing normal delivery, enhancing the overall experience and safety.

#### Tertiary health care level.

*“CandidateS-DH-D-01: Those who wore this dress are very cooperative during delivery because they are shy free. Pregnant women liked delivery in this dress.”*

*“Candidate S-DH-SN-01: Yes, along with prevention of exposure of the entire body it also helps to minimize the infection attack to both pregnant women and newborn.”*

*“CandidateS-DH-D-01: Yes, in all labour rooms by implementing this dress leads to a uniform code system. For normal delivery this dress will be dress code. There will be different dress codes for OT and normal delivery. Everyone will understand that this is a uniform dress code and it is important and should be implemented in all labour rooms.”*

#### Secondary health care level.

*“Candidate S-CHC-SN-01: Yes, I think this dress will benefit the healthcare delivery system because it minimizes the infections to both mother and newborn.”*

*“Candidate NE-CHCM-DC-230: It will be successful. I encourage the use of these gowns because if the labour room is not sterile, the newborns can get infections or fever. So if a mother wears the gown during delivery, it will reduce infections and help both the mother and child.”*

#### Primary health care level.

*“Candidate S-PHCP-SN-01: Yes, because the warmth for the baby during abdominal sleep was made more advanced while using this dress.”*

*“Candidate NE-PHCJ-DC-250: I think it will be helpful in many ways, especially in infection prevention which is very essential. Moreover, it will also bring uniformity when it comes to conducting deliveries.”*

### Theme: Funds

Implementing specially designed cloth dresses for normal delivery in India varies by healthcare level. Tertiary care hospitals, which already handle extensive clothing needs, require minimal additional investment, focusing mainly on incremental costs for materials and maintenance. In addition, Secondary/primary care hospitals need moderate funding for cleaning.

#### Tertiary health care level.

*“Candidate S-DH-SN-01: There is no need to have extra funding and the government will provide everything from the central drug store.”*

“*Candidate N-DH-SN-01: This dress is good. There is no requirement of any changes in it. Also, all the facilities are provided in this dress so I don’t think any additional resources are required.” “Candidate W-LT-SN-01: No need for extra funds to implement Maathru Samman pants in the health care system.”*

#### Secondary health care level.

*“Candidate S-CHC-SN-01: We don’t need any additional funds. We have sufficient available funds.”*

*“Candidate NE-CHCM-DC-230: We get funds from CM-SMS and also from JSSK that we can provide for the mother and child(below 1 year). It provides free transportation for patients who get referred to other hospitals. CM-SMS helps patients who live far from the CHC. Through this scheme, they can stay in the CHC before their active labour. In the same way, JSSK provides free delivery, free medicines and free check-ups.”*

#### Primary health care level.

*“Candidate S-PHCT-SN-01:: We will get all our requirements by putting an indent to CDS. The requirements that are not available will be attained for local purchasers by DDO.””*

*“Candidate N-PHCK-D-01: Yes, I would really appeal the government to implement these dresses in at least to the local communities because some women still believes in home deliveries due to shyness.”*

*“Candidate NE-PHCJ-DC-250: We have several types of funds which include MHIS, IPF and also JSSK funds. These are the main funding sources in labour rooms.*

*“Candidate W-PHC-2-SN-01:Government should provide funds to support the use of MSP.”*

## Discussion

In this study the doctors and nurses who are involved in conducting normal delivery using Maathru Samman Pants are interviewed. The gender distribution in labour and delivery services, with females comprising more than four fifth of healthcare providers and males one fifth, reflects a global trend where women predominantly occupy roles in maternal health. The preference for female healthcare staff by more than three fourth of the pregnant women and the occurrence of deliveries in common labour rooms with partitions reflect cultural and social norms that prioritize comfort and modesty. These findings are supported by Iravani et al., who observed similar preferences for female staff and privacy measures in different cultural settings [6]. Internationally, studies highlight similar patterns. Anspach’s research on gender and healthcare underscores the historical and cultural roots leading to a female majority in these professions, due to traditional caregiving roles [7]. In Sweden, Carter’s study on gender performances in midwifery care reveals the dominance of female practitioners in labour settings, emphasizing gender roles that align with caregiving expectations [8]. Similarly, in Ecuador, Carpio-Arias et al. note that societal norms favor female healthcare providers for maternal services, which is reflected in the gender distribution [9].

In the Indian context, the gender dynamics are consistent. Tiwari et al. discuss the gender balance in Indian healthcare, noting a significant female presence in nursing and midwifery, akin to the all-female provider scenario observed in the North region [10]. Sahay et al. highlight regional disparities in gender representation, with certain areas showing more male involvement, similar to the West’s primary level representation [11]. Kapoor et al. address gender biases in healthcare roles, emphasizing systemic factors that perpetuate female dominance in labour and delivery services [12].

The high satisfaction rates among healthcare providers with Maathru Samman Pants (MSP) at tertiary healthcare levels underscore the significant impact these garments have on improving the comfort and dignity of women during labour. International studies have highlighted similar benefits of specialized maternity attire [13–15]. The satisfaction of healthcare providers with Maathru Samman Pants (MSP) at the secondary healthcare level highlights their significant role in improving the quality of maternal care in less resource setting health care. The agreement on the benefits of MSPs in aiding body coverage, supporting legs during delivery, and maintaining privacy when lying down or walking, aligns with global findings on the importance of appropriate maternity attire in enhancing patient dignity and comfort. The positive reception of Maathru Samman Pants (MSP) at the primary healthcare level highlights their significant utility in enhancing maternity care even in low-resource health facilities. The overall agreement on their usefulness, with more than half of healthcare providers strongly agreeing and remaining agreeing, highlights the widespread acceptance of MSPs in improving childbirth experiences. Hwang et al. found that redesigning maternity gowns to better meet user needs can significantly enhance the childbirth experience by providing comfort and functionality, which aligns with the high approval ratings of MSPs for coverage and mobility [13]. In a European context, Wilson et al. emphasized the importance of functional maternity wear in ensuring patient dignity and facilitating ease of movement during labour, which reflects with the positive feedback on MSPs from healthcare providers regarding mobility and ease of examination [14]. Furthermore, a study by Bohren et al. on respectful maternity care globally advocates for innovations that enhance maternal dignity and comfort, supporting the implementation of special garments to improve childbirth experiences [15]. In India, the introduction of MSPs represents a critical advancement in respectful maternity care. This is corroborated by Ansari and Yeravdekar, who stress the importance of respectful maternity care initiatives like MSPs in mitigating issues of disrespect and abuse during childbirth [16]. Additionally, Yadav et al. found that garments play a vital role in facilitating movement and reducing discomfort, thereby contributing to a more respectful and supportive maternity care environment [17]. The positive reception of MSPs among healthcare providers illustrates MSP’s essential role in fostering respectful maternity care. By enhancing privacy, mobility, and comfort, MSPs not only improve the childbirth experience for women but also streamline the workflow for healthcare providers, making them comfortable and providing shy free maternal care practices.

The acceptability of Maathru Samman Pants (MSP) among healthcare providers across various healthcare levels highlights their crucial role in enhancing the quality of maternity care by ensuring privacy and comfort for women during labour. At the secondary healthcare level, the near complete agreement that MSPs prevent overexposure and increase family satisfaction reflects their effectiveness in maintaining dignity and privacy, which is a fundamental aspect of respectful maternity care. This aligns with international findings, such as those by Bohren et al., which emphasize the importance of protecting maternal dignity through appropriate attire [15]. Similarly, at the primary healthcare level, the strong consensus on the benefits of MSPs in preventing overexposure and maintaining a shy-free environment highlights their pivotal role in creating a supportive and respectful labour experience. This is consistent with global perspectives, where Wilson et al. advocate for functional maternity wear that enhances patient satisfaction and facilitates a positive childbirth environment [14]. The combined data across all healthcare levels, where a significant majority strongly agree on the benefits of MSPs, reinforces their acceptability and effectiveness. The recommendation of MSPs by more than three fourth of respondents at all levels signifies their perceived value in promoting privacy and comfort, ensuring dignified and respectful maternity care. The broad acceptability of MSPs among healthcare providers shows their health care worker friendliness of the dress to enhance privacy and dignity during labour. By supporting a respectful and supportive maternity care environment, MSPs contribute significantly to improving the childbirth experience for women and their birth companions.

The demand and positive perception of Maathru Samman Pants (MSPs) among healthcare providers at various levels shows their significant role in enhancing maternal care by prioritizing comfort and privacy. Internationally, similar innovations in maternity attire have been shown to improve patient satisfaction and comfort. For instance, Wilson et al. emphasize the importance of clothing that facilitates comfort and dignity during childbirth, aligning with the high ratings given to MSPs, where more than two third of secondary healthcare respondents rated them “Very Good” compared to routine dresses [14]. Furthermore, Bohren et al. highlight the global need for maternity care practices that respect patient privacy and dignity, supporting the demand for MSPs noted in the tertiary healthcare level, where almost two third of respondents indicated MSPs can be sterilized and reused, enhancing their practicality and sustainability [15]. Similarly, Hwang et al. discuss how well-designed maternity attire can significantly impact the childbirth experience, resonating with the majority agreement in the North region on the appropriateness of MSPs for labour room clothing [13]. In India, the introduction of MSPs reflects a broader commitment to respectful maternity care. MSPs are effective in maintaining privacy and dignity, which corresponds with the unanimous agreement among providers in the North region that MSPs are “Very Good” and should be recommended to all pregnant women [16]. Additionally, Yadav et al. highlight the significance of garments like MSPs in facilitating movement and reducing discomfort, further supporting the widespread demand for MSPs across healthcare levels [12]. The demand and positive reception of MSPs among healthcare providers across regions and levels illustrate their essential role in promoting respectful and supportive maternity care.

The widespread adaptability and acceptance of Maathru Samman Pants (MSP) across healthcare levels underline their critical role in promoting respectful maternity care by ensuring privacy and cultural sensitivity. Internationally, studies support the integration of such initiatives to improve maternal experiences. Bohren et al. emphasize the global need for maternity practices that respect privacy and cultural values, aligning with more than two third of tertiary healthcare providers who strongly agree MSPs are a positive initiative [15]. Similarly, Hwang et al. highlight the importance of culturally sensitive and practical maternity attire, resonating with more than three fourth of staff who prioritize cultural respect during delivery [13]. Wilson et al. discuss the significance of maintaining privacy in maternity settings, which corresponds with the high ratings for privacy in examination and labour rooms noted in the survey [12]. In India, the adoption of MSPs reflects a commitment to enhancing respectful maternity care. MSPs’ effectiveness in maintaining privacy and respecting cultural norms, supported by the majority of secondary healthcare respondents who emphasize cultural respect as crucial [18]. Ansari and Yeravdekar advocate for initiatives like MSPs that enhance privacy and align with organizational and cultural goals, as seen in the primary healthcare level where majority rated privacy positively [13]. Yadav et al. further corroborate the importance of garments like MSPs in facilitating culturally sensitive care, aligning with more than two third of primary healthcare staff who strongly agree on MSPs’ positive impact [9]. The adaptability of MSPs across different healthcare levels and regions highlights their essential role in fostering respectful and culturally sensitive maternity care.

The practicability of Maathru Samman Pants (MSP) across various healthcare levels demonstrates their significant advantages in enhancing maternity care. The ability to sterilize and reuse MSPs multiple times, as reported by almost three fourth of health care providers, highlights their economic and environmental benefits, aligning with global practices that emphasize sustainability in healthcare [11]. The reuse capability of MSPs, noted to be 11–20 times by more than half of healthcare providers, further supports their practicality and cost-effectiveness in maternity settings. One of the critical benefits of MSPs is their role in infection control, with three fourths of healthcare providers agreeing that MSPs, combined with clean practices, help decrease infections. This aligns with international standards for infection control in maternity care, as highlighted by Wilson et al., who emphasize the importance of clean and effective clothing practices to reduce maternal and neonatal infections [12]. Comfort is another significant factor, with more than three fourth of health care workers agreeing that MSPs support mothers in various birthing positions and two third noting that they facilitate comfortable examinations and deliveries. This reflects findings by Bohren et al., who underscore the importance of comfort and flexibility in maternity attire to enhance the birthing experience [13]. Additionally, the majority of healthcare providers felt that MSPs improved communication with mothers during procedures and delivery, which is crucial for patient-centered care and aligns with respectful maternity care practices that prioritize patient comfort and communication. The positive feedback of health care providers on the practicability of MSPs across healthcare levels highlights their essential role in promoting respectful and efficient maternity care. By supporting infection control, providing comfort, and facilitating better communication, MSPs contribute significantly to improving the childbirth experience for both mothers and healthcare providers.

The introduction of Maathru Samman Pants (MSP) in maternity care settings offers numerous advantages, such as enhanced privacy, comfort, and infection prevention for pregnant women during delivery. However, like any innovation, it also presents some challenges that need to be addressed to maximize its benefits and ensure widespread acceptance. One of the primary advantages of MSPs is their ability to provide a respectful and dignified birthing experience, as they prevent unnecessary body exposure and create a “shy-free” environment, contributing to a more cooperative and comfortable delivery process. Additionally, the uniformity brought about by MSPs in maternity wards supports a standardized approach to delivery attire, which is crucial for maintaining hygiene and reducing the risk of infections. Despite these benefits, challenges of improving washing and sterilization facilities are essential for overcoming resistance to reuse, as noted by Hwang et al. in their work on the adoption of sustainable clothing practices in healthcare [13]. The reuse of MSPs offers a cost-effective and environmentally friendly alternative to disposable gowns, as they can be sterilized and reused multiple times. However, the challenges related to the cleaning process, particularly in facilities lacking proper washing equipment, need to be addressed. Providing adequate resources and support for laundry services can facilitate the successful adoption of MSPs and enhance their practicality in various healthcare settings.

The integration and expansion of Maathru Samman Pants (MSP) in labour rooms at tertiary, secondary and primary healthcare levels underscore their significant role in enhancing respectful maternity care by providing privacy, comfort, and a shy-free environment. The support for MSP use across healthcare levels highlights their acceptability and sustainability, aligning with global trends toward patient-centered maternity care. International studies, such as those by Bohren et al., emphasize the importance of maternity attire that respects privacy and reduces patient discomfort, resonating with the positive reception of MSPs among healthcare providers [15]. Similarly, Wilson et al. highlight the critical role of clothing in ensuring comfort and dignity during childbirth, which is supported by the high satisfaction rates with MSPs due to their comfort and privacy features [14]. The work by Hwang et al. further underscores the significance of well-designed maternity wear in enhancing patient experiences, aligning with the staff’s endorsement of MSPs for their practical benefits [13].

The introduction of Maathru Samman Pants (MSP) in healthcare settings has significantly improved the childbirth experience by addressing the discomfort and exposure issues associated with traditional hospital gowns or personal clothing. This shift aligns with international and Indian efforts to promote respectful maternity care. Globally, studies emphasize the need for maternity attire that prioritizes comfort, privacy, and cultural sensitivity. The importance of clothing that respects patient dignity and reduces exposure, aligning with feedback from women in various regions who found MSPs more comfortable and less exposing than traditional gowns. The significance of attire that facilitates ease of movement and comfort during childbirth, echoing the experiences of women who appreciated the practical design of MSPs. The importance of well-designed maternity wear in enhancing patient satisfaction and minimizing unnecessary exposure, reflecting the widespread approval of MSPs among women who found them a welcome change from their previous experiences. In India, the implementation of MSPs will mark a significant advancement in respectful maternity care practices. The impact of MSPs in maintaining privacy and dignity during childbirth, as evidenced by the positive feedback from women who felt more secure and comfortable.

The emphasis on respectful maternity care with the Maathru Samman pants, is crucial in this context. These initiatives aim to enhance the dignity and comfort of women during childbirth, addressing both physical and emotional needs. Respectful maternity care practices, such as those promoted through Maathru Samman, are essential in ensuring positive childbirth experiences, in any gender caregivers setting to culturally attuned to the needs of women during labour. MSP will help to bridge the gap between gender representation and patient-centered care, fostering environments where both providers and mothers feel respected and shy free. The adoption of Maathru Samman Pants (MSP) across all healthcare settings in India presents numerous compelling reasons that align with the goals of improving maternal care and ensuring respectful childbirth experiences. Firstly, MSPs enhance privacy and dignity for women during delivery, addressing a critical aspect of respectful maternity care which is essential in promoting women’s rights and well-being. Secondly, they provide comfort and a shy-free environment, which can lead to better cooperation from patients during labour, facilitating smoother delivery processes. Thirdly, by minimizing unnecessary exposure, MSPs reduce the psychological stress and embarrassment often associated with traditional delivery attire, thereby improving the overall childbirth experience.

A limitation of this study is that the focus group consisted solely of 29 female supporting staff, with no other quantitative variables examined within this group. This limits the ability to analyze the influence of other demographic or quantitative factors among supporting staff for use of MSP.

Finally, the successful integration of MSPs into healthcare settings can drive innovation and improvements in other areas of healthcare delivery, highlighting India’s leadership in advancing maternal health initiatives. By adopting MSPs, the Government of India can demonstrate its commitment to maternal health, setting a precedent for comprehensive and respectful care that can inspire global change.
